# Cardiac Calcified Amorphous Tumor in the Neonatal Period

**DOI:** 10.1155/2022/9087597

**Published:** 2022-01-10

**Authors:** Mohammad Nasir Hematian, Kamran Hessami, Maasoumeh Saleh, Abolfazl Shirdel Abdolmaleki, Shirin Torabi, Sedigheh Hantoushzadeh

**Affiliations:** ^1^Department of Perinatology and Fetal Cardiology, Tehran University of Medical Sciences, Yas Hospital, Tehran, Iran; ^2^Department of Obstetrics and Gynecology, Baylor College of Medicine, Houston, TX, USA; ^3^Department of Obstetrics and Gynecology, Tehran University of Medical Sciences, Tehran, Iran; ^4^School of Medicine, Shiraz University of Medical Sciences, Shiraz, Iran; ^5^Maternal-Fetal and Neonatal Research Center, Tehran University of Medical Sciences, Tehran, Iran

## Abstract

Calcified amorphous tumor (CAT) of the heart is a rare nonneoplastic cardiac mass that may exhibit symptoms resembling malignancy. In this report, we presented a 4-month-old male baby with repeated attacks of cyanosis and a cardiac murmur. Echocardiography revealed a tumoral noncircumscribed mass in the right atrium adhering to the interatrial septum which extends to the inferior vena cava. Cardiac exploration was carried out to excise the tumor. A histopathological study demonstrated the presence of thrombus-like tissue with extensive calcification and foreign body type giant cell reactions. After operation, the patient had an uneventful hospitalization. Although CAT is mainly diagnosed in adult patients, it should be considered in the causes of cardiac mass in the neonatal period.

## 1. Introduction

Calcified amorphous tumor (CAT) of the heart is a rare nonneoplastic cardiac mass that exhibits symptoms similar to those of malignant conditions or vegetation [1]. These calcified areas can lead to obstruction and embolization [2–4]. The etiology of CAT has not been elucidated yet. Histological features of cardiac CAT include chronic inflammation cells, calcium deposits, hyalinization, and degeneration of blood elements [2]. Thus, CAT is an intracavitary cardiac mass based on the endocardium which is microscopically composed of depositions of calcium in the nodular form in the context of chronic inflammation surrounded by amorphous fibrous material. CAT follows a benign clinical course. While its pathogenesis is obscure, some authors have speculated that mural thrombi may be the origin of CAT [1]. The diagnosis of CAT is dependent on surgical resection and histopathological examination. Almost all CAT cases are diagnosed in adult patients [1–3]. Cardiac CAT is extremely rare in newborns. Hereby, we present a case of a mass in the right atrium which was pathologically diagnosed to be CAT.

## 2. Case Report

The patient was born at 28 weeks of gestation by C/S due to placental abruption with a weight of 1020 g. The patient was admitted to the neonatal intensive care unit (NICU) with respiratory distress. Umbilical catheterization was done for IV access and TPN. Blood and urine cultures were obtained which were negative. The umbilical catheter was removed due to a high risk of infection at its site. The infant was discharged after 3 months of hospitalization. Over the 2 weeks after discharge, the infant developed cyanotic attacks and a murmur. Because of the auscultation of a cardiac murmur on physical examination and an echocardiogram revealing a cardiac mass, the infant was referred to the children′s medical center for further cardiac evaluation.

**Figure 1:**
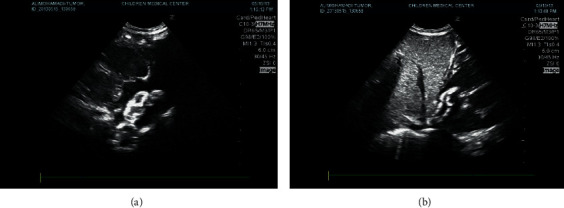
2D transthoracic, subcostal view demonstrating the echogenic mass (arrow head) attached to the interatrium septum (a) and extension to the IVC (b).

The patient's weight on readmission was 2000 g. A physical examination showed normal pulses in all extremities and both carotid arteries. His respiratory rate was 45 breaths per minute with slight dyspnea. Transcutaneous oxygen saturation in the ambient air was 95%. No splenomegaly and hepatomegaly were detected. His left arm arterial pressure was 87/56 mm Hg (mean arterial pressure was 67 mm Hg). Electrocardiographic yielded no abnormal findings. A chest X-ray showed cardiomegaly with a cardiothoracic ratio of 0.8 (normal value = 0.60) and normal pulmonary vascular markings. The laboratory tests revealed metabolic acidosis (pH = 7.29) and bilirubinemia (3.43 to 6.68 mg/dL) without direct hyperbilirubinemia. The patient was at the risk of ABO incompatibility, but Coombs direct test was negative. No anemia and leukopenia (white blood cells = 7990/mm^3^, neutrophils = 2724/mm^3^, hemoglobin = 15.1 mg/dL, hematocrit = 45%, and platelets = 316 000/mm^3^) were present. Fasting blood glucose, level of serum electrolytes, and liver enzymes were in the normal range for his age. We did not detect any coagulation abnormalities. We ruled out systemic bacterial septicemia by three consecutive negative blood cultures and normal CRP values. Serum alpha-fetoprotein of 7000 ng/ml was mildly elevated for his age. His lupus anticoagulant screening was negative. IgM serology tests for cytomegalovirus, toxoplasmosis, and rubella were negative. Brain and abdominal ultrasonography were normal. An electrocardiogram demonstrated normal sinus rhythm with right axis deviation in the normal range of age. The initial echocardiogram showed a mass in the right atrium which can cause cyanosis due to right-to-left shunt via foramen ovale, and also a large patent ductus arteriosus was identified. Transthoracic echocardiography showed a right atrial mass (2.5 × 0.5 cm) with a single nonhomogeneous focal calcification (Figure 1). With consideration of a probable diagnosis of myxoma, the mass was removed by cardiac exploration. A classic median sternotomy was performed. An extracorporeal circulation circuit was installed, and then a right atriotomy was conducted for the excision of the tumor. The PDA was also ligated. The size of the removed calcified mass was about 2.6 × 0.5 cm with multiple sites of attachment to the right atrial wall and septum and extending into the inferior vena cava (IVC). Histopathological examination showed a thrombus-like mass composed of eosinophilic amorphous fibrinous material and multiple areas of calcification. Neutrophils and cell debris were noted focally. Foreign body type giant cell reactions and collections of histiocyte-like cells with merging eosinophilic cytoplasm were also observed. There was a delicate capillary network at the periphery surrounded by dense fibroblastic cells. Multiple sections did not reveal any foci of “myxomatous” tissue. Based on the histopathological findings and clinical manifestations, a diagnosis of cardiac calcified amorphous tumor (cardiac CAT) was confirmed. Postoperative hospitalization of the patient was uneventful. No complications had occurred to the patient during an 8-month period of follow-up. The patient has been doing well during the follow-up.

## 3. Discussion

Primary tumors of the heart like atrial myxomas are rare, but nonneoplastic cardiac masses such as intramural thrombi or vegetation can mimic the features of neoplastic disorders. Regardless of the type of the masses, because there is potential risk of embolization or obstruction and also the importance of accurate diagnosis and therapy, making an excision of the cardiac lesions can be required [3, 5–7].

Introduced in 1997 for the first time [1], CAT was defined as the deposition of calcium on an amorphous fibrinous stroma with an inflammatory background and the degeneration of blood elements. Initially it was regarded as a calcified thrombus, but then it was recognized as a rare primary tumor with a benign nature. Lamination and the presence of hemosiderin differentiate organizing thrombus from CAT [1].

The size of CAT varies in different individuals and any part of the cardiac chambers can be invaded with the left atrium being the least involved cardiac region [8]. The majority of CAT cases are detected as sessile growth lesions, but pedunculated lesions have also been rarely reported [9, 10]. Reports of relapse after incomplete surgical removal [11] and calcification persistence at the origin site after complete removal [1] have also been published.

Like other cardiac masses, dyspnea, syncope, and embolism-related symptoms are the main presentations of cardiac CAT which are very unspecific. These symptoms of CAT may also be seen in thrombi, emboli, vegetation, and other cardiac tumors [12]. Histologically, CAT cases have been mistaken for tumoral calcinosis, calcified tuberculomas, calcified myxomas, or rhabdomyosarcomas [13]. For precise diagnosis of CAT out of its numerous differential diagnoses, clinical, histological, and imaging findings must be taken into consideration altogether [14].

The pathogenesis of cardiac CAT is not thoroughly understood yet. Some studies have suggested that cardiac CAT is an organized and calcified mural thrombus which is supported by the fact that in some cases, there is an association between the presence of predisposing factors of thrombosis and cardiac CAT [1]. Some other authors have speculated that abnormal calcium-phosphorus metabolism as in hemodialysis or Alport syndrome patients has been involved in the development of cardiac CAT. Patients with collagen vascular diseases or abnormal coagulation profiles have also been diagnosed with cardiac CAT. In some cases, the presence of anti-dsDNA antibodies, anticardiolipin antibodies, and deficiencies of protein S and protein C have been reported [1, 11]. However, the definite pathogenesis of cardiac CAT is still unknown.

Assessment of thrombosis as the possible origin of the tumor in our patient was not feasible because there was no evidence suggestive of perinatal cardiovascular abnormality in our patient. Other suggested factors including coagulation factors, autoantibodies, and kidney function were all normal in our patient. Hence, future studies are needed for evaluation of the perinatal period of patients suffering from cardiac CAT to identify the exact origin of the tumor.

## Data Availability

No data were used to support this study.

## References

[1] Reynolds C., Tazelaar H. D., Edwards W. D. (1997). Calcified amorphous tumor of the heart (cardiac CAT). *Human Pathology*.

[2] Vaideeswar P., Karunamurthy A., Patwardhan A. M., Hira P., Raut A. R. (2010). Cardiac calcified amorphous tumor. *Journal of Cardiac Surgery*.

[3] Gutiérrez-Barrios A., Muriel-Cueto P., Lancho-Novillo C., Sancho-Jaldón M. (2008). Calcified amorphous tumor of the heart. *Revista Española de Cardiología (English Edition)*.

[4] Lin Y.-C., Tsai Y.-T., Tsai C.-S. (2011). Calcified amorphous tumor of left atrium. *The Journal of Thoracic and Cardiovascular Surgery*.

[5] Viscardi F., Errico G., Schiavo N., Biban P., Mazzucco A., Luciani G. B. (2009). Familial fetal-type rhabdomyoma of the tricuspid valve in the neonate: malignant course for a benign disease. *The Journal of Thoracic and Cardiovascular Surgery*.

[6] Flynn A., Mukherjee G. (2009). Calcified amorphous tumor of the heart. *Indian Journal of Pathology and Microbiology*.

[7] Tsuchihashi K., Nozawa A., Marusaki S. (1999). Mobile intracardiac calcinosis: a new risk of thromboembolism in patients with haemodialysed end stage renal disease. *Heart*.

[8] Habib A., Friedman P. A., Cooper L. T., Suleiman M., Asirvatham S. J. (2010). Cardiac calcified amorphous tumor in a patient presenting for ventricular tachycardia ablation: intracardiac echocardiogram diagnosis and management. *Journal of Interventional Cardiac Electrophysiology*.

[9] Greaney L., Chaubey S., Pomplun S., St Joseph E., Monaghan M., Wendler O. (2011). Calcified amorphous tumour of the heart: presentation of a rare case operated using minimal access cardiac surgery. *Case Reports*.

[10] de Hemptinne Q., Bar J.-P., de Canniere D., Unger P. (2015). Swinging cardiac calcified amorphous tumour arising from a calcified mitral annulus in a patient with normal renal function. *Case Reports*.

[11] Fealey M. E., Edwards W. D., Reynolds C. A., Pellikka P. A., Dearani J. A. (2007). Recurrent cardiac calcific amorphous tumor: the CAT had a kitten. *Cardiovascular Pathology*.

[12] Kubota H., Fujioka Y., Yoshino H. (2010). Cardiac swinging calcified amorphous tumors in end-stage renal failure patients. *The Annals of Thoracic Surgery*.

[13] Bhag G., Kumar G., Sahai K., Arora H. S., Sharma V. K. (2018). Cardiac calcified amorphous tumor in a newborn. *The Annals of Thoracic Surgery*.

[14] Sousa J. S. d., Tanamati C., Marcial M. B., Stolf N. A. G. (2011). Calcified amorphous tumor of the heart: case report. *Revista Brasileira de Cirurgia Cardiovascular*.

